# Multifunctional Peptides from Spanish Dry-Cured Pork Ham: Endothelial Responses and Molecular Modeling Studies

**DOI:** 10.3390/ijms20174204

**Published:** 2019-08-28

**Authors:** Sara María Martínez-Sánchez, Horacio Pérez-Sánchez, José Antonio Gabaldón, José Abellán-Alemán, Silvia Montoro-García

**Affiliations:** 1 Laboratorio de Cultivo Celular, Facultad de Ciencias de la Salud, UCAM Universidad Católica San Antonio de Murcia, Campus de los Jerónimos s/n, Guadalupe 30107, Murcia, Spain; 2 Departamento Tecnología de la Alimentación y Nutrición, UCAM Universidad Católica San Antonio de Murcia, Campus de los Jerónimos s/n, Guadalupe 30107, Murcia, Spain; 3 Bioinformatics and High Performance Computing Research Group (BIO-HPC), Computer Engineering Department, Universidad Católica de Murcia (UCAM), Guadalupe 30107, Murcia, Spain; 4 Cátedra de Riesgo Cardiovascular, UCAM Universidad Católica San Antonio de Murcia, Campus de los Jerónimos s/n, Guadalupe 30107, Murcia, Spain

**Keywords:** bioactive peptides, inflammation, NF-κB, dry-cured pork ham, angiotensin I converting enzyme, endothelial dysfunction, molecular blind docking

## Abstract

Food peptides contain a very wide range of diversified structures, which explains their diverse range of functional activities. Proatherogenic endothelium is related to vasoconstriction, inflammation, and oxidative stress. In this line, four synthetic bioactive peptides from dry-cured pork ham, previously identified according to their Angiotensin I Converting Enzyme (ACE) inhibitory capacity and high bioavailability, were tested. Among them, KPVAAP displayed an estimated IC_50_ of 59.22 µM for human ACE inhibition, and docking simulations demonstrated the consistency of the noncompetitive binding with the protein. The addition of synthetic peptides to human endothelial cells significantly prevents the expression of genes related to endothelial dysfunction and inflammation (eNOS, ICAM-1, VCAM-1, IL-6) and lowers NF-κB activation (all *p* < 0.05). In silico dockings showed that the four bioactive peptides interact with the regulatory subunit NEMO of the NF-κB transcription factor at the same site as other characterized inhibitors (CC2-LZ region). This is the first study linking experimental and computational approaches that shows NF-κB to be the target of biopeptides of food origin. These multifunctional peptides from dry-cured pork ham make them good candidates for further research into their therapeutic or preventive use to attenuate the inflammatory atherosclerotic process.

## 1. Introduction

The endothelium is an important regulator of vascular homeostasis and an essential part of the cardiovascular (CV) system, participating in all aspects of pathophysiological processes such as muscular tone, inflammation, thrombosis, or vascular wall remodeling [1]. Tumor necrosis factor-α (TNF-α) is a potent proinflammatory cytokine that triggers the canonical activation of the transcription nuclear factor-κB (NF-κB) and the endothelial gene expression of adhesion molecules, cytokines, and fibrinolytic proteins among others [2]. NF-κB is crucially involved in the pathogenesis of inflammatory diseases and represents a target for treatment. The canonical pathway is strictly regulated and involves various steps including the phosphorylation, ubiquitination, and degradation of the IκB kinase (IKK) complex, which leads to the nuclear translocation of the p50 and p65 subunits of NF-κB [3]. Small molecules such as peptides have been shown to bind with high affinity protein kinases, phosphatases [4] and inhibit ubiquitination which could explain their potential as NF-κB inhibitors [5].

Levels of inflammatory biomarkers are increased in common pathological conditions such as hypertension, hyperlipidemia, physical inactivity, among others [6]. In this last respect, beneficial dietary habits, which include anti-inflammatory and antioxidant foods, have gained in popularity for the prevention of cardiovascular diseases (CVD). Many experimental studies have documented that small sequence peptides are released during gastrointestinal digestion, food processing, and the microbial proteolysis of proteins [7]. Depending on the sequence of amino acids, these bioactive peptides (BP) can exhibit different biological activities [8], mainly through pathways that are still not clearly understood. Bioactive peptides have attracted a lot of scientific interest due to their wide range of biofunctional properties [9]. The inhibition of Angiotensin I-converting enzyme (ACE) is a widely studied effect of BP from pork meat [10], and many of these BP show high bioavailability [11]. However, few mechanistic studies have looked at possible functional properties other than ACE inhibition. In a previous clinical study, our group suggested a link between the regular consumption of dry-cured pork ham with its characteristic BP and an improvement in the inflammatory status [12].

The present study aims to confirm a robust cause and effect relationship between BP from dry-cured pork ham and beneficial physiological effects related to CV health in humans. For that purpose, changes in human ACE activity, endothelial dysfunction gene expression, NF-κB activation, oxidative and apoptotic markers were tested. In addition, molecular modelling was used to establish novel peptide-NF-κB interactions at the molecular level, which has not been attempted before. The current findings suggest that BP from dry-cured pork ham bind to the NEMO subunit of the IKK complex and might suppress the NF-κB-dependent gene expression in vitro.

## 2. Results

### 2.1. Peptides with Human in Vitro ACE Inhibitory Activity

The inhibitory activity of human overexpressed endothelial ACE was assayed in transfected cells (Figure S1), with the results expressed as IC_50_ (Table 1). By HPLC it was revealed that BP1 (KPVAAP) is a potent inhibitor of ACE activity with an estimated IC_50_ of 59.22 µM, making it 16 times more effective than BP2 and BP4 with an estimated IC_50_ > 1000 µM. BP3 also displayed a high IC_50_ of 485 μM. The initial linear dose-response pointed to the higher ACE inhibitory activity of BP1 and BP3 but was not directly comparable to the inhibitory effect of captopril on a weight-basis (IC_50_ below 10 μM).

### 2.2. Peptides Penetrate in the Catalytic Active Site of ACE

**Figure 1:**
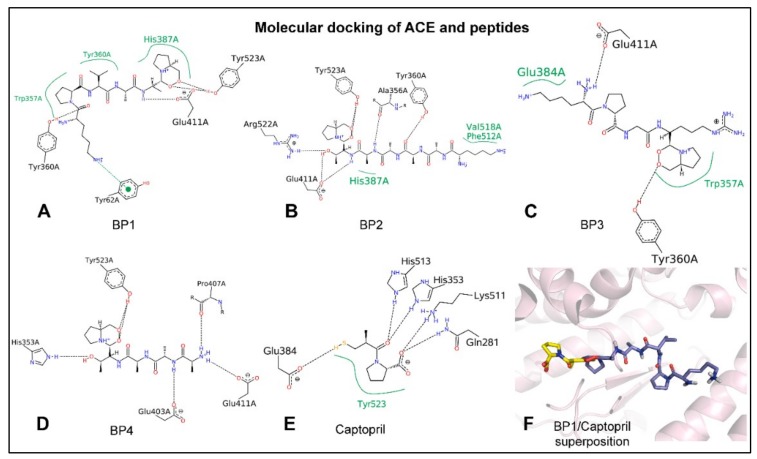
Depiction (in 2D) of the molecular docking between catalytic residues from the active site of ACE (PDB: 4APJ) and the bioactive peptides. (**A**) Peptide 1 (BP1) KPVAAP, (**B**) Peptide 2 (BP2) KAAAATP, (**C**) Peptide 3 (BP3) KPGRP, and (**D**) Peptide 4 (BP4) AAATP. (**E**) Captopril. (**F**) The superposition (in 3D) of BP1 (in purple) and captopril (in yellow) in stick representation. Continuous green lines represent hydrophobic interactions, while black dashed lines show hydrogen bonds. The absence of hydrophobic stabilization in the case of BP4 (**D**) might explain its high IC_50_ value. Moreover, BP2 (**B**) showed small hydrophobic interaction areas compared with BP1 (**A**) and BP3 (**C**), which contained voluminous hydrophobic groups.

Docking simulations were carried out (Figure 1) to provide insight at atomic level into the interactions established between the different peptides, captopril, and the active site of human ACE (PDB: 4APJ) [13]. The four peptides (BP1, BP2, BP3, BP4) and captopril were able to bind to the protease with interaction scores of −8.1, −7.1, −8.2, −7.1, and −8.2 kcal/mol, respectively (Table 1). The area of the hydrophobic interactions and/or the establishment of hydrogen bonds (Figure 1) depend on their distinct sequences and resulting interaction patterns. Figure 1F shows that BP1 occupied the S1 and S2 subsites (near the active site), while the commercial antihypertensive drug, captopril, was found deeper inside the active site of ACE.

### 2.3. Peptide Effects in Inflammatory Conditions

#### 2.3.1. Peptides Affect Gene Expression in Inflammatory Conditions

**Figure 2:**
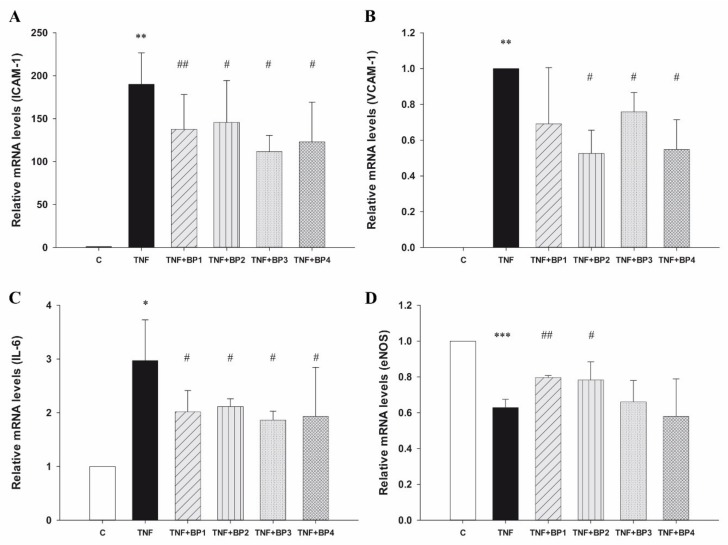
Relative mRNA expression of (**A**) ICAM-1, (**B**) VCAM-1, (**C**) IL-6, (**D**) eNOS in Ea.hy926 cells after treatment with 100 ng/mL TNF-α and 300 µM synthetic peptides. Data shown represent averaged values of three independent experiments. The asterisks *, **, and *** indicate statistically significant differences compared with unstimulated cells (*p* < 0.05, 0.01, or 0.001, respectively). #, ##, or ### indicate statistically significant differences compared with stimulated cells (*p* < 0.05, 0.01, or 0.001, respectively).

To define the stimulatory conditions in which endothelial cells express high levels of adhesion and inflammatory markers, EA.hy926 cells, a well-established endothelial model of large vessel endothelium, were treated with 10–200 ng/mL of the prototypic inflammatory cytokine TNF-α [14,15]. To develop an optimal inflammatory cell model with TNF-α-stimulated cells and investigate its response to anti-inflammatory BP, doses (10, 50, and 100 ng/mL) and durations (6 h and 12 h) of TNF-α treatment were optimized. Since the increase in protein expression reached a maximum at 6 h, further experiments were performed in EA.hy926 cells after 6 h of treatment with 100 ng/mL TNF-α. Incubation with TNF-α rapidly increased the transcription of ICAM-1, VCAM-1, and IL-6 genes and decreased eNOS expression compared with control conditions (all *p* < 0.05) (Figure 2). However, the addition of 300 µM synthetic peptides significantly prevented ICAM-1 mRNA overexpression in TNF-α activated cells (*p* < 0.05) (Figure 2A). In addition, BP2, BP3, and BP4 also downregulated the expression of VCAM-1 mRNA after stimulation with TNF-α (*p* = 0.01; *p* = 0.03; *p* = 0.02, respectively) (Figure 2B). Treatment with the four peptides produced a similar effect on IL-6 mRNA expression (all *p* < 0.05) (Figure 2C), while eNOS mRNA expression only recovered after treatment with BP1 and BP2 (*p* = 0.0003 and *p* = 0.04, respectively) (Figure 2D). Importantly, the treatment using 300 μM synthetic peptides alone—without stimulation—did not significantly alter the cell expression of these four genes or their viability compared to control conditions (Figures S2 and S3A, respectively).

#### 2.3.2. Peptides Affect Protein Expression in Inflammatory Conditions

The corresponding flow cytometry (FACS) analysis pointed to an increase in ICAM-1 mean fluorescence intensity (MFI) on the cell surface after TNF-α stimulation (*p* = 0.0003) (Table 2). The coincubation of synthetic peptides (BP1, BP2, and BP4) and TNF-α resulted in a reduced ICAM-1 surface density compared to TNF-α activated cells (all *p* < 0.05) (Table 2, Figure S4B). Despite the higher number of VCAM-1 positive cells, the surface staining of VCAM-1 was hardly detectable even after TNF-α stimulation and no effect was found after the addition of synthetic peptides to the activated cells (Figure S4C). Peptide treatment alone did not impair the MFI of adhesion molecules compared to control conditions.

The intracellular protein expression of ICAM-1 in the same conditions as above was confirmed by western blot (Figure S4D). Treatment with three of the four peptides (BP2, BP3 and BP4) consistently resulted in a lower production of ICAM-1 protein, compared with that produced after TNF-α stimulation (*p* < 0.01) (Figure S4D). No intracellular VCAM-1 was detected in these cells using the same approach.

### 2.4. Effect of Peptides in Oxidative Conditions

#### 2.4.1. Peptides Do Not Affect Cell Viability and Apoptosis after Treatment with H_2_O_2_

To explore the role of synthetic peptides in oxidative conditions, the endothelial cell function was impaired by means of high H_2_O_2_ concentrations, as it has been previously reported [16,17]. Treatment with synthetic peptides did not improve cell viability after the H2O_2_ treatment (Figure S3B). Moreover, 300 µM H_2_O_2_ caused 10% apoptosis (AnV+, PI−cells) and 18% necrosis (AnV+, PI+ cells), and BPs were unable to improve this cytotoxic effect (Table S1).

#### 2.4.2. Peptides Slightly Affect the Oxidative Status

The mRNA expression of the redox enzymes, IL-6 and BAX, were analyzed by RT-PCR, after 16 h of preincubation with synthetic peptides followed by 24 h with 300 µM H_2_O_2_ (Figure S5). In the presence of H_2_O_2_, the expression of eNOS increased 3.3-fold (*p* = 0.01). Pretreatment with synthetic peptides did not affect the expression of the redox enzymes in oxidative conditions. Consistent with this, H_2_O_2_ upregulated IL-6 mRNA expression 1.84-fold (*p* = 0.001), independently of the presence of synthetic peptides. The proapoptotic gene BAX showed a 1.5-fold increase in expression after H_2_O_2_ treatment (*p* = 0.02) and synthetic peptides did not modify the previously obtained expression levels.

Subsequently, the effect of synthetic peptides on protein carbonylation was determined by immunoblotting assay, which showed that the protein carbonylation levels significantly increased after only 30 min of H_2_O_2_ treatment. In the presence of BP1 and BP3, the basal oxidative status was maintained (Figure S6) (all *p* < 0.05). The overall protein carbonylation level of unstimulated cells was like that seen in the presence of peptides alone, except in the presence of BP3, when it was even lower (Figure S6).

### 2.5. Peptides Decrease NF-κB Activity

**Figure 3:**
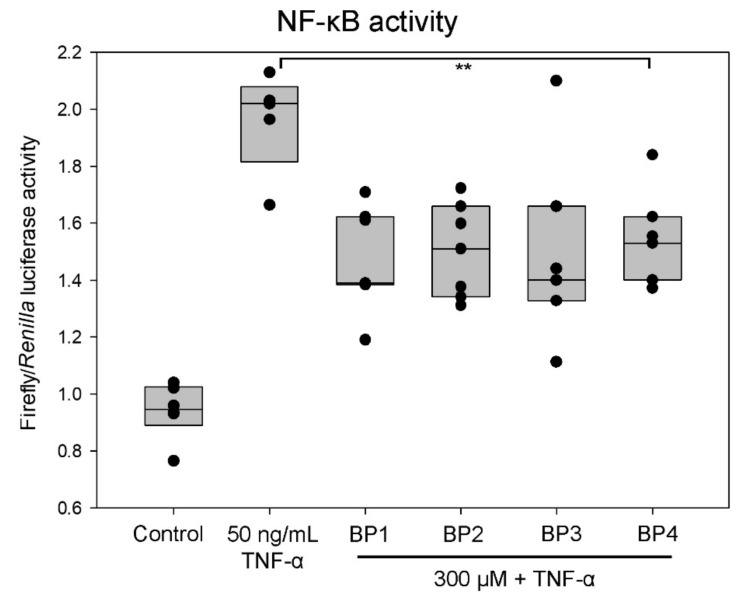
Activation of NF-κB after treatment with 50 ng/mL TNF-α and 300 µM synthetic peptides. Ea.hy926 cells were transfected with NF-κB:Luc together with the pRL-CMV (10:1) reporter vectors. The normalized luciferase activity (firefly/Renilla) was measured using the Dual-Luciferase Reporter Assay in extracts from control, TNF-α, and synthetic peptide-treated cells. Upper and lower bars of box plots represent the 25th and 75th percentiles, respectively. Median is represented as the bar inside the box plot. The data shown represent values of three independent experiments (dots). The asterisks ** indicate statistically significant differences compared with stimulated cells (*p* < 0.015). The BP treatment prevented TNF-α-induced proinflammatory NF-ĸB activation by 25%.

To evaluate whether the synthetic peptides could modulate the NF-κB biological activity, a luciferase reporter assay was carried out, using the Renilla reporter as a specificity control and indicator of cell proliferation [18]. In this study, NF-B activity was induced at a lower concentration of TNF-α (50 ng/mL) because of the high sensitivity of the approach. The normalized firefly/Renilla luciferase activity was reduced by around 25% of the activity when cells were pretreated with the BP1 (*p* = 0.002), BP2 (*p* = 0.003), and BP3 (*p* = 0.003) synthetic peptides (Figure 3). The NF-κB luciferase activity was also repressed by BP4 to a lesser degree (*p* = 0.012). The basal activity for NF-κB was not inhibited by synthetic peptides without TNF-α stimulation (data not shown). These results suggest that synthetic peptides may target events needed for NF-κB activation, rather than other transcription factor activations.

### 2.6. Peptides Bind to the Subunit NEMO

**Figure 4:**
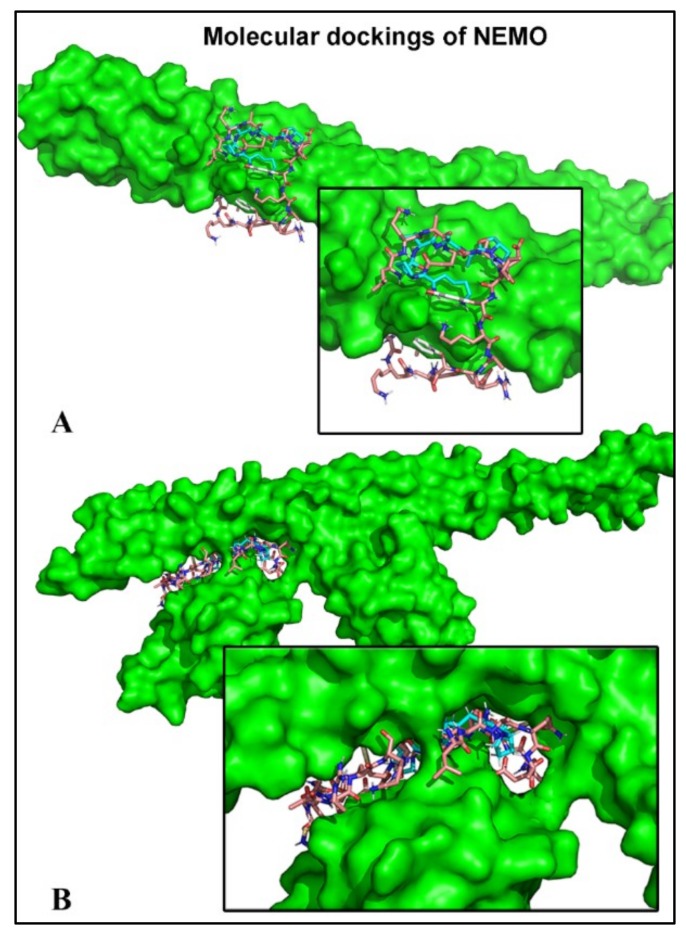
Blind docking of the UBI peptide and BP1 in the CCL-Z2 binding domain of NEMO. (**A**) 4BNW without linked ubiquitins. (**B**) 3JSV complexed with Lys 63-linked poly-ubiquitin. The superposition of UBI peptide and BP1 (in blue) is shown. In silico docking calculations support the potential binding of the four distinct BP, which could impair IKK recruitment through competition with the Lys63-linked poly-ubiquitin.

Blind docking approaches were used to model peptide binding interactions with the regulatory subunit of NF-κB, NEMO. In silico docking calculations support the potential binding of the four distinct peptides around the residue Glu315, which is crucial for functional assessment and could impair IKK recruitment through competition with the Lys63-linked poly-Ub (Table 3) [19]. The theoretical interactions of these BP and the NF-κB inhibitors are shown (Table 3A,B). Interestingly, BP1–4 efficiently bound to the same site as the anthraquinone derivative of the natural emodin (iNUB) (Table 3A,B). Indeed, the UBI peptide and BP1–4 were all found in the ubiquitin binding motifs of 4BNW and 3JSV (Table 3A,B, respectively and Figure 4). When the CCL-Z2 region displayed Lys63-linked poly-Ub (PDB: 3JSV), peptide binding appeared to be weaker due to the absence of hydrogen bonds, except in the case of BP2 (Table 3B).

## 3. Discussion

Numerous reports have identified stable peptides with high ACE-inhibitory activity from pork dry-cured ham after gastrointestinal digestion [11,20]. Four of them were synthetized chemically (KPVAAP, KAAAATP, KPGRP, and AAATP) for the current experimental studies with human endothelial cells. The synthetic peptides were much more effective ACE inhibitors when purified rabbit ACE was used in vitro: KPVAAP (BP1, 12.37 μM), KAAAATP (BP2, 25.64 μM), KPGRP (BP3, 67.02 μM), and AAATP (BP4, 100 μM) [21], an inconsistency that might be due to the presence of other proteins in the endothelium lysate extract. In both assays, BP1 was the most potent peptide and BP4 the least potent.

Docking simulations were carried out with human ACE and the different BP in order to explain the respective IC_50_ values. Molecular modelling showed that BP1, BP2, and BP3 are stabilized by both hydrogen and hydrophobic interactions with at least one residue of the three pockets, consistent with the typical competitive inhibition model. BP1 and BP2, particularly, established hydrophobic interactions with HIS387 and hydrogen bonds with TYR360, TYR523, and GLU411, while BP3 reached hydrophobic stability with GLU384 and TRP357 and formed hydrogen bonds with TYR360 and GLU411. Moreover, BP4 only established hydrogen bonds with GLU411, HIS353, and TYR523. The absence of hydrophobic stabilization in the case of BP4 might explain its high IC_50_ value. BP2 showed small hydrophobic interaction areas compared with BP1 and BP3, both of which contained voluminous hydrophobic groups. Therefore, the stability of BP2 seems to be comparable to that of BP4. Besides, captopril formed up to five H-bonds with key residues within the active site (GLU384, TYR523, and HIS353 residues). BP1 was found to occupy the S1 and S2 subsites (near the active site) but was not located inside the active site of ACE, in contrast to captopril. Therefore, we concluded that, structurally, BP are noncompetitive inhibitors of ACE so their IC_50_ were much higher compared to captopril. The present data are of special interest for predicting and understanding mechanisms of action of BP and may also be of help for predicting new biomolecules before the relevant assays are carried out.

Apart from its ACE inhibitory capacity, food-derived peptides have been shown to display a wide range of functional activities over the CV system. In fact, multifunctional peptides interfere with more than one biological pathway, such as NO production, oxidative stress, and inflammation. The Ea.hy926 cell line demonstrates highly differentiated functions of human vascular endothelium, such as expression of inflammation cytokines (e.g., IL-6) and adhesive markers (ICAM-1 and VCAM-1) [14]. Our interest in the use of an in vitro model of endothelial dysfunction was to further understand the regulatory effects of the BP treatment in inflammatory conditions. In fact, endothelial NOS was found slightly overexpressed in the presence of these synthetic peptides, which could also be beneficial for attenuating endothelial dysfunction. Similarly, a decrease in inflammatory markers (IL-6 and eNOS) has also been reported with the milk-derived peptides VPP and IPP in spontaneous hypertensive rats using DNA microarray [22]. Egg-derived IRW peptide also inhibited the TNF-α-induced increase of adhesive molecules [23]. Moreover, BP from milk impaired human endothelial–monocyte interactions by inhibiting the expression of VCAM-1, ICAM-1, and E-selectin [24]. Regarding the surface expression of proteins, their measurement strongly depends on the detachment method. The overall surface expression of ICAM-1 was found to be trypsin-resistant, while VCAM-1 was much more sensitive to trypsin degradation [25]. This fact could explain the different results on VCAM-1 gene and protein expression here reported. No mechanisms that might regulate these inhibitions have been identified, but some studies suggest that NF-kB is the main target [24]. These results are in line with a previous clinical study carried out in humans, where the regular intake of dry-cured ham containing the currently described BP lowered plasmatic IL-6, P-selectin, and MCP-1 levels but did not alter plasmatic VCAM-1 [12]. All this evidence supports the hypothesis that the dual ability of these BP to modulate adhesive markers and eNOS might be due to the NF-κB interaction.

On the other hand, oxidative stress and inflammation are closely related pathophysiological processes, one of which can be easily induced by another. Likewise, oxidative stress can activate the NF-κB pathway [26]. In the current study, the simultaneous use of synthetic peptides and H_2_O_2_ was ineffective at reducing cell death, indicating that these peptides do not target apoptotic mechanisms of action (for instance, caspases or BAX proteins). Besides, the in vitro antioxidant capacity of these peptides [27] was not supported by the current results since the redox enzymes remained unaltered and the carbonylation protein approach was not sensitive enough to confirm the reduction of reactive oxygen species. 

Nonetheless, it is apparent from the current data that TNF-α-induced NF-κB activation is sharply attenuated in human endothelial cells in the presence of these peptides from dry-cured pork ham. Several studies have also demonstrated the anti-inflammatory role of specific peptides [28]. However, the research efforts were limited to the biological effects and the action mechanisms were not deduced [29]. In our case, blind docking approaches were used to model peptide binding interactions with the regulatory subunit of NF-κB, NEMO. Recent studies have shown that direct binding of NEMO to linear polyubiquitin (poly-Ub) chains in the TNF-α signaling pathway is crucial for kinase (IKKα/β) recruitment and further NF-κB activation [30,31].

Furthermore, optineurin and small molecules (anthraquinone derivatives and peptides) have previously been suggested to negatively regulate TNF-α-induced NF-κB activation by competing with this CC2-LZ region for Lys63-linked poly-Ub [32,33]. We further found that the BP used experimentally could bind to the same region as a competitive peptide, the UBI peptide, specifically designed to interfere with the coiled interfaces of NEMO [33]. The targeting of IKKβ by dry-cured pork ham peptides was unexpected and suggests the potential regulatory role of the canonical pathway of NF-κB activation identified in the in vitro approaches.

The CCL-Z2 region interacts with Lys63-linked poly-Ub chains with relatively low affinity, which could facilitate the disruption by small molecular compounds, such as peptides [19]. Therefore, the possibility that the BP under study have a biological effect due to their interaction with NEMO before and/or after polyubiquitin binding (PDB: 4BWN and 3JSV, respectively) is very likely. Since both interactions are possible, our data do not determine whether these peptides impair ubiquitin binding by competing at the site or modulating it after TNF-α stimulation. Nonetheless, these data must be interpreted with caution because the inhibitory concentration and bioavailability of these multifunctional BP are still unknown, thereby limiting clinical anti-inflammatory therapeutic strategies. At this point, it is important to mention that this study does not attempt to use attainable eating levels of bioactive peptides (300 μM) but provides the conceptual and operational tools for investigating the sites of action of BP in the context of inflammatory pathological mechanisms. The study may lead to better understanding of the effects of food-derived BP as ACE-inhibitors, the findings being of indirect clinical relevance.

## 4. Material and Methods

### 4.1. Peptides

Spanish dry-cured ham has been reported as a good source of bioactive peptides with potent ACE inhibitory activity in vitro [21]. Four of these identified peptides were synthesized chemically by GenScript Corporation (Piscataway, NJ, USA) at the highest purity certified using liquid-chromatography mass spectrometry (LC-MS) analysis for the current experimental approaches with human endothelial cells. The sequence and protein origin of the peptides are shown in Table 1.

### 4.2. Cell Culture

EA.hy926 cells, the hybrid human umbilical vein endothelial cell line, were obtained from the American Type Culture Collection (ATCC^®^ CRL2922™, Rockville, MD, USA). Cells were cultured in high glucose Dulbecco’s Modified Eagle’s Medium (DMEM), containing 10% heat-inactivated fetal bovine serum (FBS, Biowest, Riverside, CA, USA) and 50 U/mL of penicillin and 50 µg/mL streptomycin (Sigma Aldrich Chemical Co., Saint Louis, MO, USA). Cells were grown in 5% CO_2_ in a humidified air incubator at 37 °C. Subculture was performed when 90% confluence was reached.

### 4.3. Human ACE Inhibition Assay

To overexpress the ACE enzyme, EAhy926 cells were transiently transfected with a human Ace ORF mammalian expression plasmid (Sinobiological, Beijing, China) using trans-it X2 reagent (Myrus^®^, Madison, WI, USA). The resultant ACE had a terminal peptide Myc and the efficiency of transfection was checked by immunofluorescence, using an antimyc antibody (Sigma Aldrich Chemical Co., Saint Louis, MO, USA) (Figure S1).

Cells were lysed using 200 µL M-PER^®^ Mammalian Protein Extraction Reagent (ThermoFisher, Waltham, MA, USA). Human ACE inhibitory activity of the four chemically synthetized peptides was tested in vitro. Briefly, 20 µg of lysate (protein) was incubated for one hour at 37 °C with peptides concentrations (50–1000 µM) containing 5 mM Hippuryl Histidyl Leucine (HHL) (Sigma Aldrich Chemical Co., Saint Louis, USA) as substrate. Captopril (10 µM) was used as the positive control of inhibition for the assay conditions. The HHL was transformed into hippuric acid (HA), which was detected by high-performance liquid chromatography (HPLC, Shimadzu, Kioto, Japan) in a C18 column (Teknokroma, Madrid, Spain). The mobile phase was composed of solvent A, 0.05% trifluoroacetic in water; and solvent B, 100% acetonitrile. The ratio of solvent A/solvent B was 7/3, and the flow rate was 1.5 mL/min. The elute was analyzed at a wavelength of 214 nm, which is the maximum absorbance of HA, and the column temperature was maintained at 25 °C. The IC_50_ value (the concentration of inhibitor resulting in a 50% reduction of ACE activity) was calculated by regression analysis from the ACE inhibition curve obtained with increasing amounts of synthetic peptides.

### 4.4. TNF-α and H_2_O_2_ Stimulation

EA.hy926 were seeded in six-well plates at 0.25 × 10^6^ cells/well in DMEM supplemented with 5% FBS. Cells were treated with 300 µM of each peptide for 16 h. Then, 100 ng/mL TNF-α or 300 µM H_2_O_2_ were added for an additional 6 h or 24 h, respectively.

### 4.5. Quantitative RT-PCR

Total RNA was extracted from EA.hy926 cells using 300 µL Trisure™ (Bioline, Taunton, MA, USA) reagent and Direct-zol™ RNA MiniPrep (Zymo Research Irvine, Irvine, CA, USA) according to the manufacturer’s protocol. Total RNA was reverse-transcribed into complementary DNA (Sensifast cDNATM Synthesis kit, Bioline, Taunton, MA, USA). The mRNA levels of the target genes were quantified by RT-PCR using SensiFAST SYBER Hi-ROX Kit (Bioline, Taunton, MA, USA) with StepOnePlus Real-Time PCR System (Applied Biosystems, Foster City, CA, USA). Briefly, 5 µL of 1:5 diluted cDNA was added to the qPCR reaction containing 10 μL 2X SensiFAST Mix and 400 nM of each primer in a total volume of 20 μL. 

Specific and validated primers for human glyceraldehyde-3-phosphate dehydrogenase (GADPH), intercellular adhesion marker-1 (ICAM-1), vascular cell adhesion marker-1 (VCAM-1), endothelial nitric oxide synthase (eNOS), interleukin 6 (IL-6), catalase, SOD (Super Oxide Dismutase), NADPH (Nicotinamide Adenine Dinucleotide Phosphate oxidase) and Bax (proapoptosis regulator) genes were used (Sigma-Aldrich Chemical Co., Saint Louis, MO, USA). 

The relative mRNA expression of the genes of interest was represented by:

2^(−ΔΔCT) = [CT_(gene of interest)_ − CT_(GADPH)_]test − [CT_(gene interest)_ − CT_(GADPH)_]control.

The relative quantification of gene expression was determined by the comparative fold change 2^ΔΔCT method [34]. An average value of each target gene after GAPDH normalization at the time point showing highest expression was used as a calibrator to determine the relative levels in the rest of the experimental conditions. All the assays were performed in triplicate. Each qPCR reaction had three replicates.

### 4.6. Flow Cytometry

To measure adhesion molecule content in human endothelial cells by flow cytometry, the cell monolayer was detached and fixed before immunofluorescence labelling [25]. The following antibodies were used per sample: 20 µL anti-ICAM-1/CD54-phycoerythrin (PE) (clone HA58, BD Biosciences, Franklin Lakes, NJ, USA) and 5 µL anti-VCAM-1/CD106-PerCP-Cy5.5 (clone 51–10C9; BD Biosciences, San Jose, CA, USA), according to the manufacturer’s protocol. Stained cells (10,000 events) were examined by flow cytometry (FACS Calibur, Becton Dickinson, Mountain View, CA, USA) in a simultaneous two-color analysis with FL2 (PE) and FL3 (PerCP-Cy5.5) channels. Markers were set according to the negative controls to quantify the percentage of positively stained cells. Mean Fluorescence Intensity (MFI) was calculated for each antigen (MFI of total stained cells-MFI of negative control cells). 

ApoScreen Annexin V Apoptosis Kit-FITC (Southern Biotech, Birmingham, AL, USA) was used to detect the apoptosis rate. After the co-incubation of synthetic peptides and 300 µM H_2_O_2_, 0.4 × 10^6^ cells were detached and washed in cold PBS, then resuspended in 100 µL Annexin V binding buffer and treated following the manufacturer’s protocol. Annexin V-fluorescein isothiocyanate (FITC) binding was assessed in FL1 channel simultaneously with propidium iodide (FL2 channel). This test discriminates among intact cells (FITC−/PI−), apoptotic cells (FITC+/PI−), and necrotic cells (FITC+/PI+). Assays were performed in triplicate (Table S1).

### 4.7. NF-κB Activity

The dual luciferase assay has been widely used in cell lines to rapidly and accurately determine the activity of the promoter of NF-κB. The transfected vector, pNF-κB:Luc, carries the luciferase gene under the control of three synthetic copies of the κB consensus of the immunoglobulin κ-chain promoter cloned in the BamHI site located upstream of the conalbumin transcription start site. This construct, together with the pRL-CMV, were kindly provided by Dr. Cayuela-Fuentes (Hospital Universitario Virgen de la Arrixaca, Murcia, Spain) [18].

For luciferase reporter assays, 80,000 cells/well were seeded in 24-well plates overnight, followed by cotransfection with 0.5 μg pNF-κB:Luc/pRL-CMV at a ratio of 10:1 using 1.5 μL trans-it X2 reagent (Myrus^®^, Madison, WI, USA). The transfection media was changed 6 h after transfection by complete growth medium. The following day, cells were treated with the synthetic peptides for 16 h and then with 50 ng/mL TNF-α for 6 h. NF-κB-dependent firefly luciferase activity and NF-κB-independent Renilla luciferase activity were assessed using Dual-Luciferase^®^ Reporter Assay System (Promega, Madison, WI, USA) in a Luminometer Optocomp I (MGM Instruments). Data were normalized to the amounts of Renilla luciferase activities, according to the manufacturer´s protocol.

### 4.8. Molecular Modelling

In order to obtain detailed information at atomic level about the interactions between the different peptide molecules, inhibitors and the human proteins, molecular modelling studies were carried out. 

A representative X-ray crystal structure for human ACE (PDB code 4APJ) was chosen, and its full atom model for the docking simulation was prepared. Docking simulations were chosen as the most adequate molecular modelling technique for the ACE study, since they efficiently predict at a reasonable computing cost electrostatic, van der Waals, hydrogen bond and hydrophobic interactions between interacting ligands and protein [35]. Partial charges and hydrogens were added with Autodock Tools [36]. The studied peptide molecules and characterized inhibitors were built manually using Pymol [37].

Two representative X-ray crystal structures of the NF-κB essential modulator, NEMO, were retrieved from the PDB database (PDB: 4BWN and 3JSV), and their full atom models for the docking simulations were prepared. The sequence for the UBI peptide was extracted from the work of Chiaravalli et al. [32] and the structure from the anthraquinone derivative, iNUB, was retrieved from the work of Vincendeau et al. [38]. In order to determine in which part of the CC2-LZ region of NEMO the different ligands interact, a blind docking approach [39] where the researcher does not define any preferred interaction spot [40] was followed, where multiple docking runs started around the geometric centers of all residues. A histogram with the resulting distribution of binding energies and their structural clusters of poses was generated. Each individual docking simulation was performed with the Autodock Vina software AUTODOCK using default configuration parameters [41]. The size of the grid box was set to extend 120 Å in each direction from the geometric center of each individual docking simulation. The docking score produced by Autodock Vina was taken as the predicted value of the ligand binding energy. Only the top-ranked poses were used for structural and energy analyses. The scoring function from Vina considers the Lennard-Jones term (LJ), hydrogen bonds (H-bonds), electrostatic interactions, hydrophobic stabilization, entropic penalty due to the number of rotatable bonds, and the internal energy of the ligand.

### 4.9. Statistical Analyses

Data were expressed as mean ± standard deviation (SD) of three determinations. A Student’s *t*-test was used to compare the differences between the mean of two groups. Statistical analyses were performed with SPSS 21.0. Statistical significance was considered at *p* < 0.05.

## 5. Conclusions

The overall goal of the study was to identify which specific mechanisms of dry-cured pork ham peptides are operative in inflammatory pathways and, as a result, to identify the most promising targets for functional food development. Reported molecular modelling results explained the rationale of their anti-inflammatory activity for the first time. The amino acid sequence of these inhibitory peptides may also form the basis for the design of analogues with therapeutic potential. Further, the multifunctional peptides characterized here may herald an important avenue in food and pharmacological research.

## Figures and Tables

**Table 1:** Angiotensin I-converting enzyme (ACE) inhibitory peptides derived from dry-cured pork ham: sequence, estimated IC_50_ value and interaction score. Captopril (10 µM) inhibition was used to represent 100% inhibition of ACE activity under the assay conditions.

Bioactive Peptide	Sequence	Source of Protein	IC_50_ (µM)	Interaction Score
PEPTIDE 1 (BP1)	KPVAAP	Myosin-XV	59.22 ± 3.8	−8.1
PEPTIDE 2 (BP2)	KAAAATP	PR domain Zinc Finger Protein 2	>1000	−7.1
PEPTIDE 3 (BP3)	KPGRP	Titin	485.50 ± 43.47	−8.2
PEPTIDE 4 (BP4)	AAATP	PR domain Zinc Finger Protein 2	>1000	−7.1

Data from three independent experiments are expressed as mean ± SD. Specific ACE activity was expressed as µmoles of substrate HHL converted to the product HA per unit of time and normalized for protein content (units per microgram of protein).

**Table 2:** Mean fluorescence intensity (MFI) of ICAM-1 surface expression in inflammatory conditions measured by flow cytometry.

	ICAM-1 MFI	*p* Value
Control	71.36 ± 8.31	
TNFα	1271.91 ± 158.54	0.0003
TNFα + BP1	1151.37 ± 59.53	0.03
TNFα + BP2	1064.42 ± 32.02	0.009
TNFα + BP3	1203.01 ± 279. 89	0.09
TNFα + BP4	926.01 ± 152.46	0.04

ICAM-1: Intercellular Adhesion Molecule-1; VCAM-1 Vascular Adhesion Molecule-1; TNF-α: Tumor Necrosis Factor α; BP: Bioactive peptide. Data from six independent experiments are expressed as mean ± SD. TNF-α stimulated cells are compared to control conditions, and preincubations with 300 µM synthetic peptides are compared to 100 ng/mL TNF-α alone.

**Table 3:** Blind docking simulation results showing the main residues involved in the interactions with the docked BP and compounds. (**A**) Docking to 4BWN PDB. (**B**) Docking to 3JSV PDB. Common residues are marked in bold.

**A**	**Ligand**	**PDB Code**	**Hydrogen Bonds**	**Hydrophobic**
	BP1	4BWN	ALA314A, GLU315A, GLN317A	ALA314A, ALA318A
	BP2	4BWN	ASP311A, GLN313A, ALA314A, GLU315A, GLN317A	PHE312B
	BP3	4BWN	ASP311A, GLU315A, LYS326B, GLU327B	ALA314A, LYS325A
	BP4	4BWN	ALA314A, GLN317A	
	iNUB	4BWN	LYS326B, GLU327B	LYS321A, ALA323B
	UBI peptide	4BWN	GLU315B, GLN317A, ARG319A, GLU320A, LYS321A,	GLN317A, ALA318A, GLU320A, LYS321A, ALA323A
**B**	**Ligand**	**PDB Code**	**Hydrogen Bonds**	**Hydrophobic**
	BP1	3JSV	THR55A, SER57A, ASP58A, ASN60A	
	BP2	3JSV	ASP39A, GLY76A	PRO37A
	BP3	3JSV	LYS63B, SER65B	
	BP4	3JSV	GLU18B, LYS63B, ARG74A, ARG312D	
	iNUB	3JSV	ARG312D	ALA311C, LEU315C, VAL316D, LYS319D
	UBI peptide	3JSV	GLU24A, ASN25A, ASP39A, ARG42A, LEU50A, ASP52A, GLY53A, GLN62B, GLU64B	THR22A, GLU 24A, PRO38A, GLY53A, THR55A, GLU64B

iNUB is an anthraquinone derivative (8-hydroxy-9,10-dioxo-9,10-dihydro-1-anthracenyl 2-phenylcyclopropanecarboxylate). UBI peptide: LKAQADIYKARFQAERHAREK (21 residues).

## Supplementary Materials

Supplementary materials can be found at https://www.mdpi.com/1422-0067/20/17/4204/s1.

## Abbreviations

ACE	Angiotensin I-Converting Enzyme
BP	Bioactive Peptide
BSA	Bovine Serum Albumin
CV	Cardiovascular
CVD	Cardiovascular Disease
DMEM	Dulbecco Modified Eagle Medium
FBS	Fetal Bovine Serum
HA	Hippuric Acid
HHL	Hippuryl Histidyl Leucine
ICAM-1	Intercellular Adhesion Molecule-1
IKK	IκB kinase
IL-6	Interleukin-6
NF-κB	Nuclear Factor-κB
OD	Optic Density
PBS	Phosphate Buffer Saline
PVDF	Polyvinylidene Difluoride
VCAM-1	Vascular Adhesion Molecule-1
TNF- α	Tumor Necrosis Factor-α
