# Supplementary material for: Multifunctional Peptides from Spanish Dry-Cured Pork Ham: Endothelial Responses and Molecular Modeling Studies
Source: Int J Mol Sci. 2019 Aug 28;20(17):4204. doi: 10.3390/ijms20174204 (PMC6747274; doi:10.3390/ijms20174204)
Supplement: Supplementary file 1 [file ijms-20-04204-s001.pdf]

Supplementary Material and Methods

1.1. Human ACE Inhibition Assay

Before transfection, the endothelial ACE expression level was very low and the product of the enzymatic reaction undetectable, and so a recombinant construct encoding the Myc-Tag fused to the ACE gene was used. To overexpress the ACE enzyme, EA.hy926 cells were seeded in 24-well plates at 80,000 cells/well and transiently transfected with a human Ace ORF mammalian expression plasmid (Sinobiological, Beijing, China) using trans-it X2 reagent (Myrus®, Madison, Wisconsin, USA). The resultant ACE protein had a terminal peptide c-Myc. The efficiency of transfection was checked by immunofluorescence. Briefly, 48 h post-transfection, cells were fixed with 4% paraformaldehyde in culture medium for 15 min at room temperature, blocked with 1% bovine serum albumin (BSA) in phosphate buffer for 30 min followed by 0.2% saponin for 30 min to permeabilize the membrane. The same buffer was used to incubate (for 1 h) the primary mouse anti-c-myc (1:1000, clone 9E10, MA1-980) and the secondary donkey anti-mouse IgG (1:200, Alexa 647, A31571) (ThermoFisher Scientific Inc., Rockford, IL, USA). Finally, cells were stained with DAPI and observed with a Nikon Eclipse Ti microscope equipped with Plan Fluor objectives (20×, 40×, and 60×) and a digital Sight DS-QiMc camera (Nikon, Nikon Instruments, Tokyo, Japan) with a Z lens with spacing of 0.4 μm at 387 nm/447 nm, 472 nm/520 nm, 543 nm/593 nm, and 650 nm/668 nm filter sets (Semrock, Rochester, NY, USA), and the NIS-Elements AR software (Nikon).

1.2. Quantitative RT-PCR

The mRNA levels of the target genes (ICAM-1, VCAM-1, IL-6, eNOS, SOD, catalase, NADPH, BAX) were quantified by RT-PCR using SensiFAST SYBER Hi-ROX Kit (Bioline, Taunton, USA) with StepOnePlus Real-Time PCR System (Applied Biosystems, Foster City, CA, USA) using specific and validated primers (Sigma-Aldrich Chemical Co., Saint Louis, MO, USA).

1.3. Cell Viability

The tetrazolium assay (MTT) was used to estimate the cell viability in the presence of synthetic peptides (up to 600 μM) and 300 μM H_2O_2. Exponentially growing cells were seeded into a 96-well plate at a density of 1×10^4 cells/well. After 48 h of treatment, cells were incubated with MTT solution at a final concentration of 1 mg/mL for 4 h at 37 °C. The formazan crystals formed in the intact cells were dissolved in 200 μL dimethyl sulfoxide (DMSO) for 30 min and the absorbance was measured in a microplate reader at 570 nm and 620 nm of reference. Results were calculated as cell viability (%) = average optic density (OD) of wells/average OD of control wells.

1.4. Western Blot Analysis

Protease inhibition cocktail (P8340 Sigma-Aldrich Chemical Co., Saint Louis, MO, USA), and phosphatase inhibitors (NaF and Na_3VO_4) were added to the cell protein extracts. The homogenates were centrifuged (13,000× *g*, 20 min, 4 °C) and the protein concentrations in the supernatants were determined by the BioRad Protein Assay (BioRad, Hercules, CA, USA) using BSA as standard (Sigma-Aldrich Chemical Co., Saint Louis, USA). Protein extracts (10 μg) were separated by 8% sodium dodecyl sulfate-polyacrylamide gel electrophoresis (SDS-PAGE), transferred onto polyvinylidene difluoride (PVDF) membranes using the Trans-Blot® Turbo™ Transfer System (Bio-Rad, Hercules, CA, USA), and then identified by immunoblot analysis. Rabbit anti-ICAM-1 and VCAM-1 at a dilution of 1:1000 (Santa Cruz Biotechnology, Texas, TX, USA) were used. Mouse anti-actin (Sigma-Aldrich Chemical Co., Saint Louis, MO, USA) at a dilution of 1:5000 was used for reference.

1.5. Protein Carbonylation with Oxyblot

Protein carbonyl content is the most general and well-used biomarker of severe oxidative protein damage [1]. Cells were treated with synthetic peptides for 16 h before adding 300 μM H_2O_2 and leaving for additional 30 min. Protein carbonyl groups levels were determined by immunoblotting using the Oxyblot Protein Oxidation Detection Kit (Millipore Merck, Temecula, CA, USA) according to the manufacturer's protocol. PVDF membranes were blocked with 1% BSA in PBS–0.05% Tween 20 (*v*/*v*) (PBS-T) for 1 h at room temperature. The blots were incubated overnight at 20 °C with the polyclonal antibody. Subsequently, blots were washed (3–5 times) with PBS-T and incubated with goat anti-rabbit IgG/HRP conjugate (1:300 dilution in PBS/BSA) (Sigma-Aldrich Chemical Co., Saint Louis, MO, USA) for 1 h at room temperature. An enhanced chemiluminescence kit (Pierce ECL detection kit, ThermoFisher Scientific Inc., Rockford, IL, USA) was used for detection. To exclude nonspecific reactions, the 2,4-dinitrophenylhydrazine (DNPH) incubation step was omitted in a control experiment. In this condition, no signal was detected in the PVDF membranes (data not shown). The emitted chemiluminescent signals were detected using a digital imaging system (ImageQuant LAS500, Thermo FisherScientific Inc., Rockford, IL, USA) after automatic exposition time. Quantitation of the densitometry plots for chemiluminescence detection was carried out using ImageJ software for the analysis of 1D gels. Peaks were drawn from the distinct western-blot bands, the area were calculated and normalized to control conditions.

Supplementary Figure Legends

Table S1. Apoptotic and necrosis rate measured by FACS in oxidative conditions.

	**Apoptosis (AnV+/PI-)**		**Necrosis (AnV+/PI+)**	
	**% cells**	**P value**	**% cells**	**P value**
**Control**	0.90 ± 0.36		1.45 ± 0.24	
**H_2O_2**	10.37 ± 1.33	**0.03**	18.56 ± 3.38	**0.01**
**H_2O_2 + BP1**	14.91 ± 2.11	NS	13.86 ± 3.72	NS
**H_2O_2 + BP2**	14.47 ± 2.42	NS	19.58 ± 0.18	NS
**H_2O_2 + BP3**	15.28 ± 0.47	NS	14.08 ± 0.25	NS
**H_2O_2 + BP4**	12.16 ± 1.86	NS	18.42 ± 6.34	NS

AnV: Annexin V; PI: Propidium Iodide; BP: Bioactive peptide; NS: Nonsignificant.
Data from three independent experiments are expressed as mean ± SD.
H_2O_2 stimulated cells are compared to control conditions, and preincubations with 300 μM synthetic bioactive peptides are compared to 300 μM H_2O_2 alone.

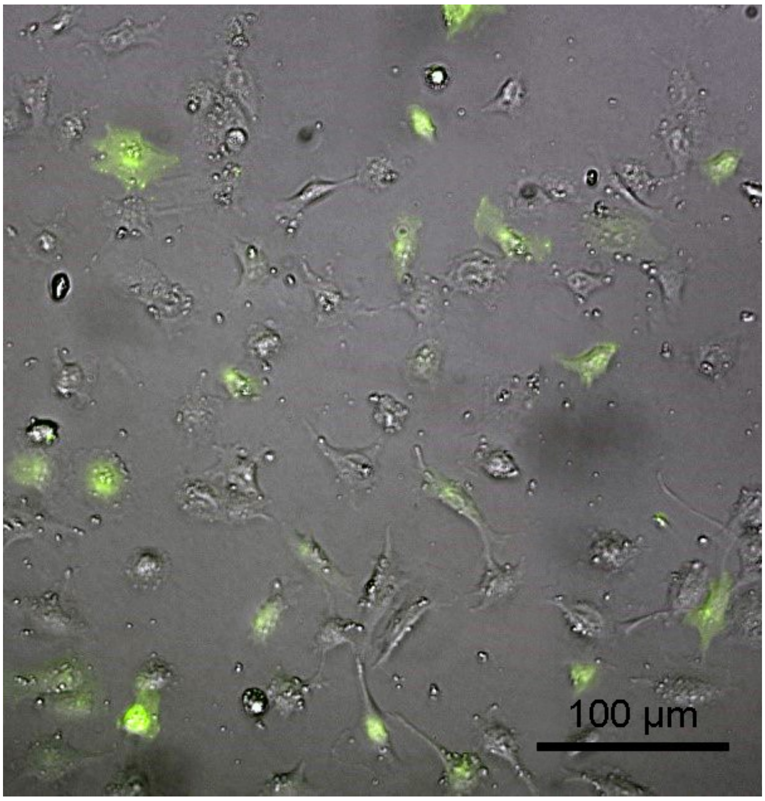

Figure S1. Martínez-Sánchez et al., 2019.

Supplementary Figure S1. Immunocytochemistry analysis of c-Myc in transfected EA.hy926 cells. 40× fluorescence and phase contrast merged images were observed by fluorescence microscopy after 48 h of transfection. The fixed cells were observed using c-Myc monoclonal antibody at a dilution of 1:1000.

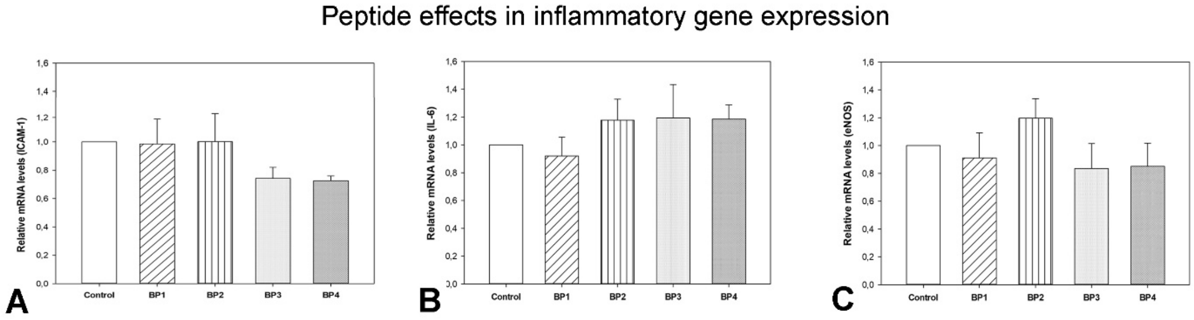

Figure S2. Martínez-Sánchez et al., 2019.

Supplementary Figure S2. Effects of synthetic bioactive peptides treatment (300 µM) alone on inflammatory gene expression. After 16 h treatment, mRNA was reverse transcribed for quantitative real-time PCR analysis of expression of A) ICAM-1, B) IL-6 and C) eNOS. VCAM-1 gene expression was undetectable. Expression was normalized to GADPH and reported as the fold-expression relative to the control group. Values shown are the mean ± SD of three independent experiments.

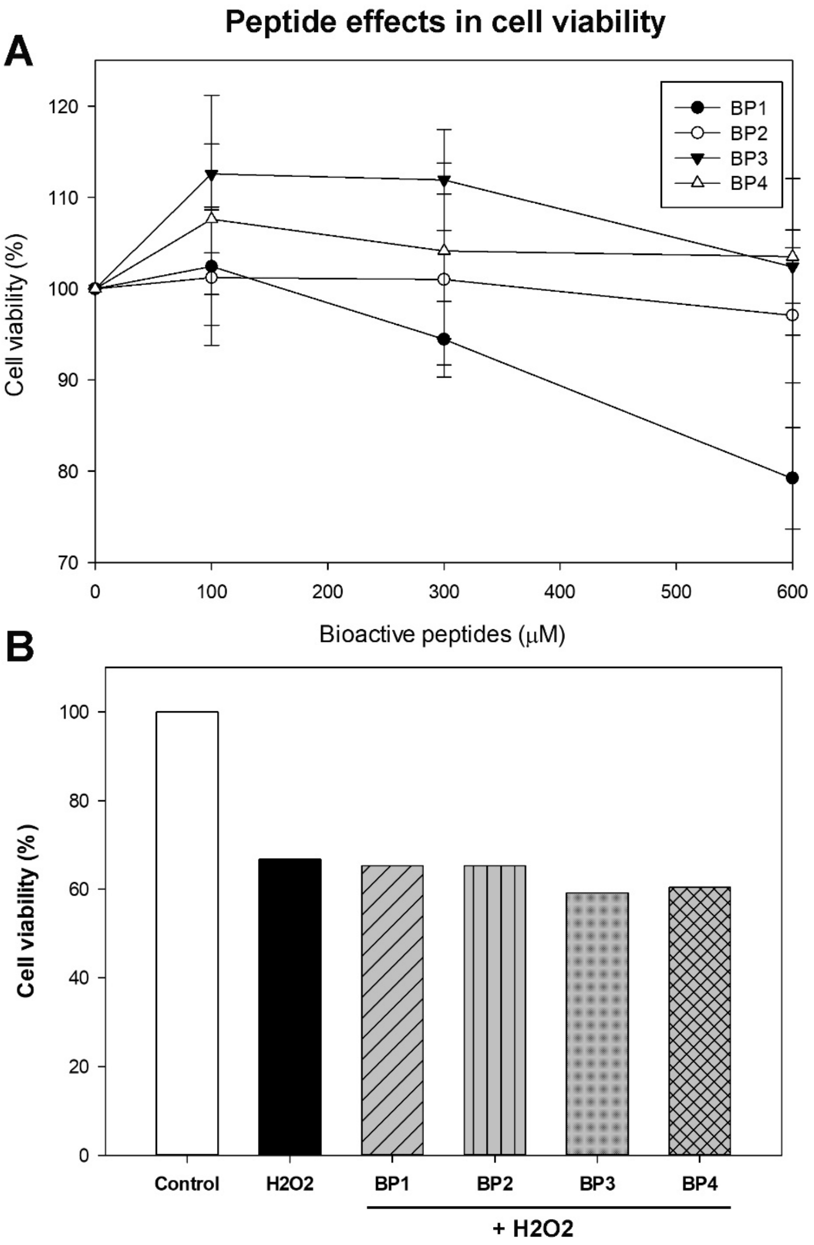

Figure S3. Martínez-Sánchez et al., 2019.

Supplementary Figure S3. Cell viability measured by MTT assay. A) In the presence of increasing concentrations of synthetic bioactive peptides (up to 600 μM). B) After preincubation with 300 μM synthetic bioactive peptides and then, 300 μM H_2O_2.

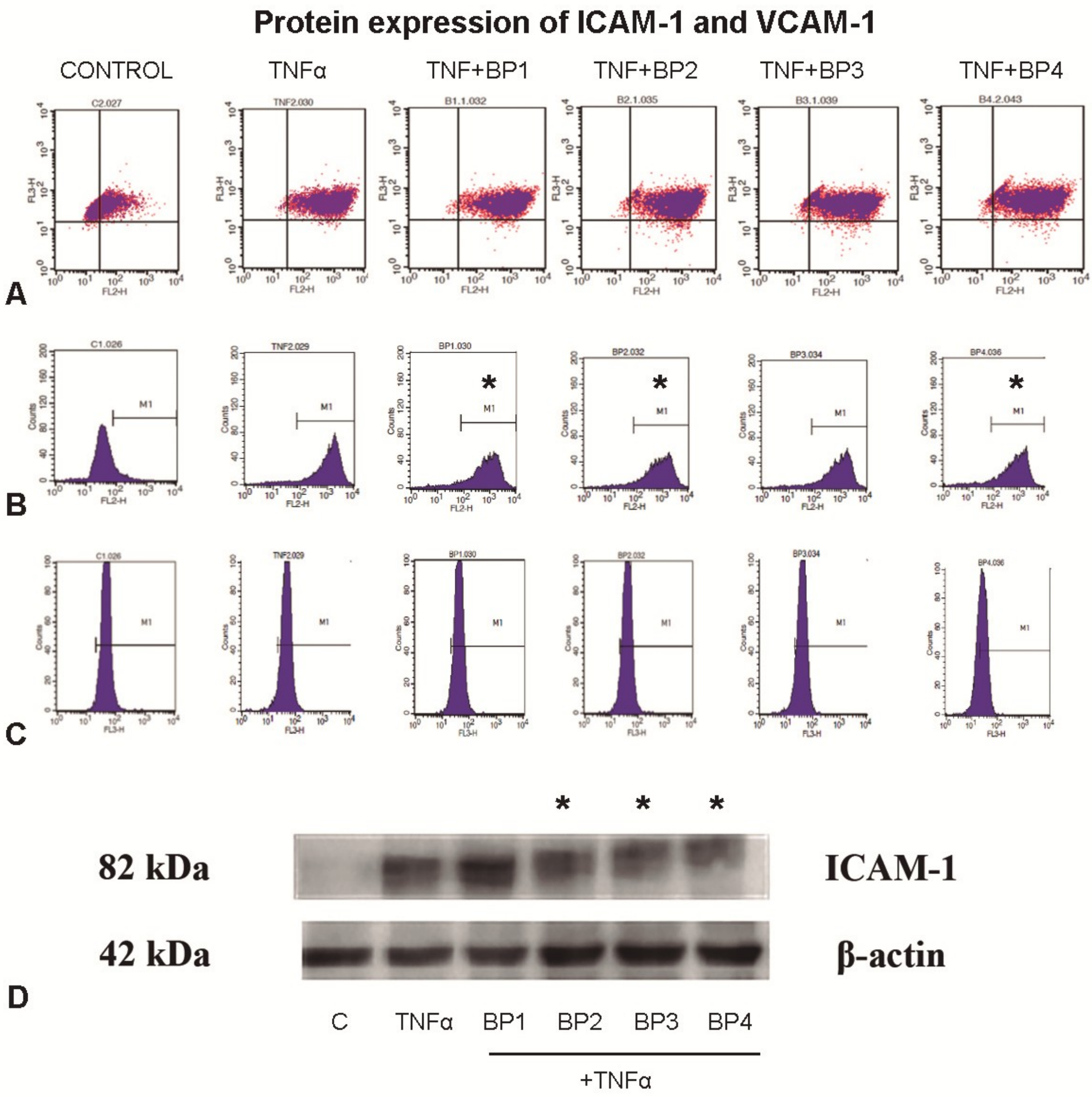

Figure S4. Martínez-Sánchez et al., 2019.

Supplementary Figure S4: Analysis of protein expression of ICAM-1 and VCAM-1 markers. **A–C)** Flow cytometric analysis. B) FL2 channel (ICAM-1); C) FL3 channel (VCAM-1). The results showed that the MFI in TNF-α-treated cells for ICAM-1 (FL2) was significantly higher than in control conditions. VCAM-1 protein expression (FL·3) was undetected even in the presence of 100 ng/mL TNF-α. **D)** Western blot analysis. Band intensity was used to measure differences in protein expression of ICAM-1 in the cell culture after the 100 ng/mL TNF-α treatment. TNF-α induced the expression of 82–85 kDa protein, corresponding to ICAM-1. Actin was used as a loading control. The asterisk * indicates a statistically significant difference compared to stimulated cells ($p < 0.05$).

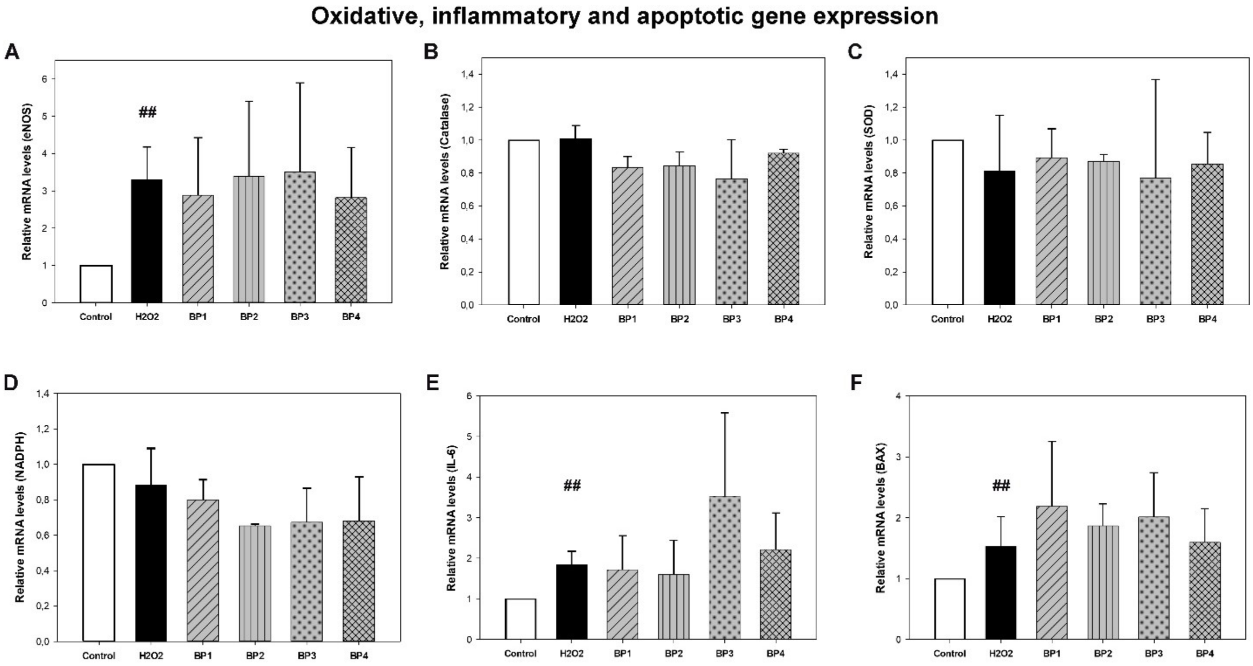

Figure S5. Martínez-Sánchez et al., 2019.

Supplementary Figure S5. Gene expression after treatment with 300 μM synthetic peptides and then 300 μM H_2O_2. Endothelial cells mRNA was reverse transcribed for quantitative real-time PCR analysis of gene expression of A) eNOS, B) Catalase, C) Super Oxide Dismutase (SOD), D) Nicotinamide Adenine Dinucleotide Phosphate oxidase (NADPH), E) Interleukin-6 (IL-6), and F) BAX. The expression was normalized to GADPH and reported as the fold-expression relative to the control condition. Values shown are the mean ± SD of three independent experiments. ## indicates a statistically significant difference compared to unstimulated cells ($p < 0.01$).

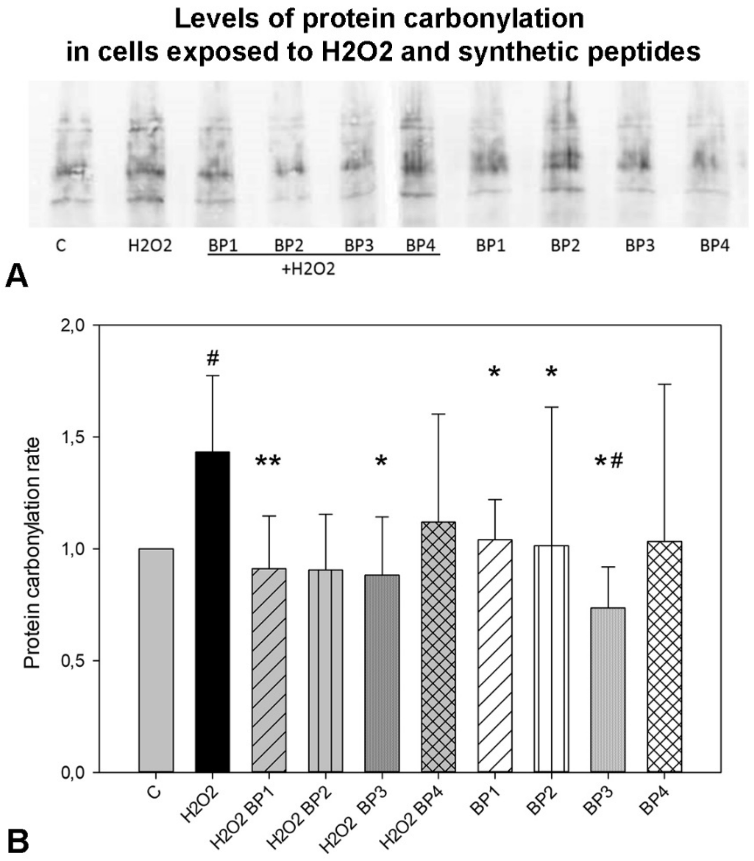

Figure S6. Martínez-Sánchez et al., 2019.

Supplementary Figure S6. Oxidized protein levels after treatment with 300 μM synthetic peptides and then 300 μM H_2O_2. **A**) Western blot. Band intensity was used to measure differences in protein carbonylation levels in the cell culture after H_2O_2 treatment. **B**) Representative blots. Data represent averaged values of triplicate measurements. # indicates a statistically significant difference from unstimulated cells (p < 0.05). The asterisks (* or **) indicate a statistically significant difference compared to stimulated cells (p < 0.05 or 0.01, respectively).

Supplemental references

1. Dalle-Donne, I.; Rossi, R.; Giustarini, D.; Milzani, A.; Colombo, R. Protein carbonyl groups as biomarkers of oxidative stress. *Clin Chim Acta* **2003**, *329*, 23–38, doi:10.1016/s0009

1
